# Excitation and coherent control of spin qudit modes in silicon carbide at room temperature

**DOI:** 10.1038/s41467-019-09429-x

**Published:** 2019-04-11

**Authors:** V. A. Soltamov, C. Kasper, A. V. Poshakinskiy, A. N. Anisimov, E. N. Mokhov, A. Sperlich, S. A. Tarasenko, P. G. Baranov, G. V. Astakhov, V. Dyakonov

**Affiliations:** 1 Ioffe Institute, St. Petersburg, 194021 Russia; 2 Experimental Physics VI, Julius-Maximilian University of Würzburg, Würzburg, 97074 Germany; 3 National Research University of Information Technologies, Mechanics and Optics, St. Petersburg, 197101 Russia; 4 Peter the Great St. Petersburg Polytechnic University, St. Petersburg, 195251 Russia

## Abstract

One of the challenges in the field of quantum sensing and information processing is to selectively address and coherently manipulate highly homogeneous qubits subject to external perturbations. Here, we present room-temperature coherent control of high-dimensional quantum bits, the so-called qudits, associated with vacancy-related spins in silicon carbide enriched with nuclear spin-free isotopes. In addition to the excitation of a spectrally narrow qudit mode at the pump frequency, several other modes are excited in the electron spin resonance spectra whose relative positions depend on the external magnetic field. We develop a theory of multipole spin dynamics and demonstrate selective quantum control of homogeneous spin packets with sub-MHz spectral resolution. Furthermore, we perform two-frequency Ramsey interferometry to demonstrate absolute dc magnetometry, which is immune to thermal noise and strain inhomogeneity.

## Abstract (web summary)

High-dimensional quantum bits advance the application of quantum sensing and information processing technologies but suffer from the low spectral selectivity and working temperature. Here the authors present the selective excitation and control of spin qudits modes based on an ensemble of silicon vacancy defects in silicon carbide at room temperature.

## Introduction

Quantum bit or qubit is a two-level system, which builds the foundation for quantum computation, simulation, communication, and sensing^1^. Quantum states of higher dimension, i.e., qutrits (*D* = 3) and especially qudits (*D* = 4 or higher), offer significant advantages. Particularly, they can provide noise-resistant quantum cryptography^2^, simplify quantum logic^3^, and improve quantum metrology^4^. Flying and solid-state qudits have been implemented on the basis of photonic chips^5^ and superconducting circuits^6^, respectively. The extension of the superconducting phase qubit to *D* = 5 were used to emulate the dynamics of single spins *S* = 1/2, 1, and 3/2, allowing a measurement of the Berry phase under 2*π* rotation and paving a way to complex quantum computational protocols and architectures. However, superconducting phase qudits operate in the millikelvin temperature range. Therefore, there is still a lack of room-temperature qudits with long coherence time.

To overcome this challenge, we have considered optical active centers in solids. Up to now, only two materials have been known to host the centers with spin and optical properties allowing coherent control and manipulation of their ground state spins by means of the optical and radiofrequency quanta at ambient condition. Namely, diamond containing negatively charged nitrogen-vacancy (NV^−^) centers and silicon carbide (SiC) containing silicon–carbon divacancies or negatively charged silicon vacancies (V_Si_) centers^7–9^. Commercially produced wafer-scale SiC possesses ideal combination of hardness, radiation stability, and thermal conductivity of diamond, with the semiconducting properties and technological maturity of silicon^10^. In addition, the polymorphism of SiC enables the tuning of magnetic and optical characteristics of the centers^11,12^.

The divacancy centers posses the triplet ground state (*S* = 1), similarly to the NV^−^ centers in diamond. They have been recently used to demonstrate a variety of prominent examples of the quantum phenomena in solids^8,13^, including the quantum entanglement in a macroscopic spin ensemble at ambient conditions^14^. Despite the unique properties, the triplet ground states of both the NV^−^ centers in diamond and divacancies in SiC impose limitations on their use as qudits.

These limitations are lifted for the centers with the *S* = 3/2 (*D* = 4) ground state, i.e., for the negatively charged silicon vacancy (V_Si_) centers in SiC. Due to stable single-photon emission in NIR range, moderate zero-field splitting in the MHz region, zero-phonon lines with high Debay–Waller factor (up to 40%), and long spin coherence times up to 20 ms at cryogenic temperatures, V_Si_ centers were proposed for various quantum applications^8,9,15–17^. However, until now these centers have been treated as a canonical *S* = 1/2 qubit system^9,15–19^.

Here, we develop a concept of operating V_Si_ centers as qudits, which can reside in multiple superpositions of four basis states in the Hilbert space with the spin projections *m*_S_ = ± 3/2, ± 1/2. Similar to atomic orbitals, an ensemble of such spin qudits is described by 15 linearly independent spherical multipoles, with three components being the spin dipole ${\cal{P}}$, five components being the spin quadrupole ${\cal{D}}$, and seven components being the spin octupole ${\cal{F}}$^20^. Examples of such multipoles and their description in terms of the spin-density matrix *ρ* are sketched in Table 1. When qudit multipoles are excited, they decay with three relaxation times: that of the spin dipole (*T*_*p*_), quadrupole (*T*_*d*_), and octupole (*T*_*f*_).

**Table 1:** Illustration of the V_Si_ spin multipoles and their mathematic representation via the basis diagonal matrices ${\cal{I}}$, ${\cal{P}}_0$, ${\cal{D}}_0$, and ${\cal{F}}_0$

We use this concept to experimentally excite the qudit modes at ambient conditions by means of two-frequency optically detected magnetic resonance (ODMR). Strikingly, we found their spectral width to be about an order of magnitude narrower than the inhomogeneous broadening of the corresponding spin resonance. By applying the Ramsey interferometry to these spin qudits, we achieve a spectral selectivity of 600 kHz and a spectral resolution of 30 kHz. As a practical consequence, we demonstrate absolute DC magnetometry insensitive to thermal noise and strain fluctuations.

We focus on the V_Si_ centers in two main hexagonal polytypes of SiC, namely 6 H and 4 H, with natural (^29^Si 4.7%, ^13^C 1.1%) and modified (^29^Si 1%, ^13^C 1.1%) isotope abundance (see the Methods section). Exemplary electron paramagnetic resonance (EPR) spectrum of the investigated 6H-^28^SiC sample is shown in Supplementary Fig. 1a.

## Results

### Visualization of the spin qudit modes

To experimentally visualize the V_Si_ spin qudit modes, we use optically detected magnetic resonance (ODMR) as described elsewhere^11,15,19,21,22^ (see also Methods and Supplementary Fig. 2). Optical pumping results in a preferential population of either the *m*_*S*_ = ± 3/2 or *m*_*S*_ = ± 1/2 states (depending on SiC polytype and the V_Si_ crystallographic site, as was shown in refs. ^9,12,20,23^). Such a spin alignment is theoretically described by the contributions to the diagonal components of the spin-density matrix *δρ*_+3/2,+3/2_ = *δρ*_−3/2,−3/2_ = −*δρ*_1/2,1/2_ = −*δρ*_−1/2,−1/2_. In the multipole decomposition of the spin density matrix, this corresponds to the appearance of the spin quadrupole ${\cal{D}}_0$ (see Table 1). Application of a strong microwave (MW) field at a fixed frequency *v*_pump_ mixes the *m*_*S*_ = ± 3/2 and *m*_*S*_ = ± 1/2 states, resulting in the excitation of other qudit modes as well. They are probed as relative difference of the spin-dependent photoluminescence ΔPL/PL, while sweeping the frequency of a second (weak) MW field *v*_probe_.

**Fig. 1:**
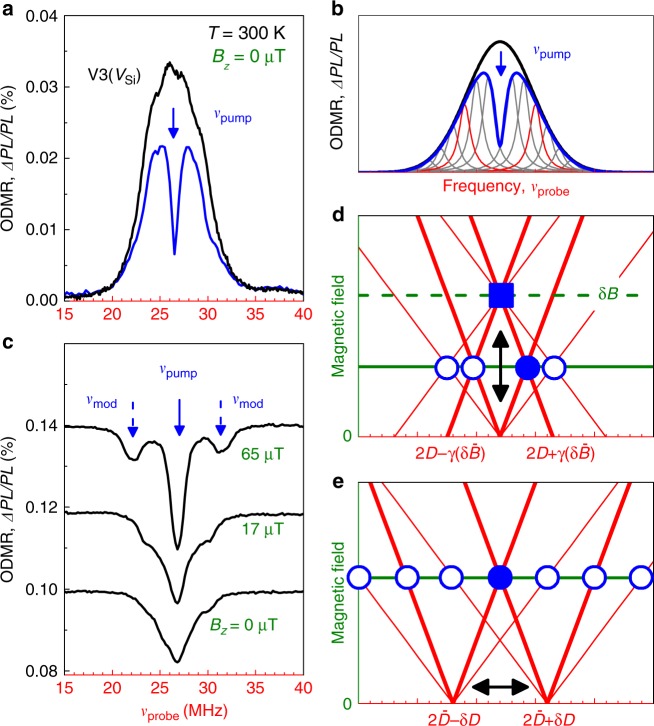
Two-frequency ODMR spectroscopy. **a** ODMR spectrum of isotopically purified 6H-SiC without and with the pump MW field at *v*_pump_ = 26.8 MHz. The pump and probe powers are *W*_probe_ = 7 dBm and *W*_pump_ = 14 dBm, respectively. **b** A schematic modeling of (**a**) by subtraction the contribution of a homogeneously broadened spin packet from the Gauss-shaped ODMR line. **c** Pump-induced changes in the ODMR spectra in different magnetic fields. The dashed arrows indicate the qudit mode frequencies. **d**, **e** The fan charts of the qudit modes as a function of the magnetic field in case when the inhomogeneous broadening is caused by magnetic fluctuations and variation for the zero-field splitting, respectively. The solid symbols correspond to the pump frequencies and the open circles correspond to the excited qudit mode frequencies. The red lines of the fan charts indicate the spectrally selected spin packets

Figure 1a shows an ODMR resonance associated with the V3(V_Si_) center in 6H-^28^SiC^23–25^. The ODMR resonance without the pump MW field (black curve) reveals the zero-field spin splitting 2*D* =  26.8 MHz between the Kramers doublets ±1/2 and ±3/2 states, whereas the sign of *D* determining the ordering of the Kramers doublets, is negative^12^. Application of a strong, pump MW field at *v*_pump_ =  26.8 MHz saturates the spin transitions, that is seen as a spectral hole burning^26,27^. To model it, we assume that the ODMR resonance is inhomogeneously broadened and agregates many homogeneous spin packets with different resonance frequencies, as schematically shown by thin lines in Fig. 1b. The pump MW field excites qudit modes in particular spin packets, which are detected as a reduction of the ODMR signal at certain frequencies. In zero magnetic field, all the excited modes are degenerated and manifest themselves as a single spectral hole. Remarkably, the spectral width of such hole can be much narrower than the inhomogeneous linewidth, and in certain cases we observe 250 kHz (Supplementary Figs. 3, 4).

The two-frequency technique enables potentially the access to the properties of spin centers with the resolution determined by the natural linewidth of individual centers. At the same time, the detected signal is much stronger and less noisy than that from a single center, which is crucial for applications.

To increase the sensitivity, we modify the detection scheme. The MW pump is now modulated on/off, and the ODMR spectrum is detected using a lock-in amplifier. The pump-induced changes of the ODMR spectrum are presented in Fig. 1c for several magnetic fields *B*_*z*_ applied along the *c*-axis of SiC. Several qudit modes *v*_mod_ are now clearly detected and their spectral positions relative to *v*_pump_ depend on *B*_*z*_.

To understand the spectrum of spin qudit modes, we analyze successively the most probable sources of inhomogeneous broadening: (i) magnetic fluctuations (for instance, due to nuclear fields) and (ii) variations of the zero-field splitting (for instance, due to local strain). The first mechanism sufficiently influences the inhomogeneous broadening of the NV^−^ center ODMR transitions in diamond, as shown in ref. ^26^. Upon application of the magnetic field along the *c*-axis, a single spin packet is characterized by four resonances shifting linearly with the magnetic filed as 2*D* ± *γB*_*z*_ and 2*D* ± 2*γB*_*z*_^28^. Here, *γ* = 28 MHz/mT is the gyromagnetic ratio. The corresponding fan chart is shown by red lines in Fig. 1d. The line thickness represents the coupling strength to the MW field. Another spin packet feels another local magnetic field *δB* and the fan chart is shifted along the vertical axis in Fig. 1d. The mean value $\delta \bar B$ defines the inhomogeneous ODMR linewidth.

Assume that the pump MW field is in resonance with one of the spin transitions of a particular spin packet (the solid circle in Fig. 1d), then the other three spin resonances of the same spin packet will be effected due to the excitation and relaxation of spin multipoles, leading to the appearance of qudit modes (the open circles in Fig. 1d). Their spectral position relative to the pump frequency is symmetric in respect to the zero-field splitting 2*D*. In case when *v*_pump_ = 2*D*, the excited qudit modes remain degenerate even when $B_z \, \ne \, 0$ (the square in Fig. 1d), which contradicts to the experimental observation of Fig. 1c. Thus, we conclude that magnetic fluctuations are not the main source of inhomogeneous broadening.

We now consider the other mechanism of broadening, caused by the variation of the zero-field splitting around the mean value $2\bar D = 26.8\,{\mathrm{MHz}}$. This mechanism is natural to expect, given that we investigate isotopically purified 6H-^28^SiC with low content of the spin-carrying ^29^Si isotopes. In this case, the fan chart of the field-dependent ODMR lines associated with different spin packets are shifted along the horizontal axis in Fig. 1e. Positions of the excited qudit modes are given by *v*_mod_ = *v*_pump_ ± *sγB*_*z*_ with *s* = 1, 2, 3, and 4. They depend on the magnetic field strength, which is in qualitative agreement with the experiment of Fig. 1c.

**Fig. 2:**
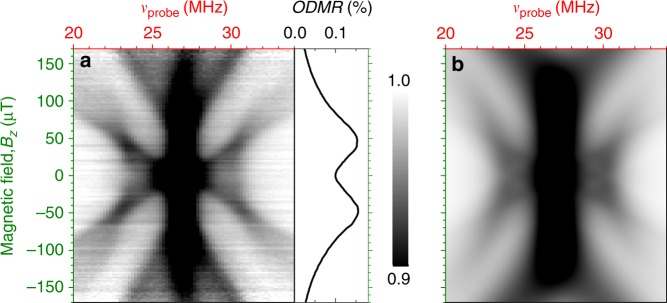
Visualization of the excited qudit modes. **a** Magnetic field evolution of the qudit modes in an inhomogeneously broadened spin ensemble. The ODMR signal is normalized for each magnetic field. The base ODMR signal is presented on the right side. **b** Calculated evolution of the spin qudit modes assuming *T*_*p*_ = 3*T*_*d*_ = 6*T*_*f*_ (see text for details)

Figure 2a visualizes the magnetic field evolution of the V3(V_Si_) spin qudit modes in 6H-^28^SiC. The observed behavior is more complex compared with the qualitative consideration above. Not all qudit modes shift linearly with *B*_*z*_, they have different strength and some of them are not even detected. To understand this behavior, we have developed a theory of qudit mode excitation in inhomogeneously broadened spin ensembles (see the Methods section).

We start from the effective spin Hamiltonian of the V_Si_ centers in a simple axial model $H = D\left( {S_z^2 - 5/4} \right) + \gamma {\mathbf{S}} \cdot {\mathbf{B}}$. In small magnetic fields that we consider, $\gamma B \ll D$, the eigenstates are given by

1 $$\begin{array}{*{20}{l}} {E_{ \pm 3/2}} \hfill & = \hfill & { + D \pm \frac{3}{2}\gamma B_z{\kern 1pt} ,} \hfill \\ {E_{ \pm 1/2}} \hfill & = \hfill & { - D \pm \frac{1}{2}\gamma \sqrt {B_z^2 + 4B_ \bot ^2} ,} \hfill \end{array}$$

where *B*_*z*_ and $B_ \bot$ are the magnetic field components parallel and perpendicular to the *c*-axis, respectively. Due to the mixing of the spin states by $B_ \bot$ and a low trigonal pyramidal local symmetry of the V_Si_ centers in SiC, all four transitions between the ±1/2 and ±3/2 spin states are allowed^28^. Their spectral positions read

2 $$\nu _{{\mathrm{mod}}}^{(s,s\prime )} = \nu _{{\mathrm{pump}}} + 3s\gamma B_z + s\prime \gamma \sqrt {B_z^2 + 4B_ \bot ^2} ,$$

where *s*, *s*′ = 0, ±1. Equation (2) describes the spectral hole burning at *v*_pump_ (*s*, *s*′ = 0) and 8 qudit modes.

We now analyze the strength of the qudit modes coupling to the MW field and how excitations between these modes are transfered. At room temperature, when the energy of thermally excited phonons is much higher than the spin splitting, one can neglect the anisotropy of spin relaxation and consider that all spin multipoles relax independently. If spin relaxation occurs due to fluctuating magnetic fields, only the transitions with Δ*m*_*S*_ = ±1 are allowed, as had also been assumed in the earlier works^15^. Application of this constraint to the matrix of spin relaxation, see Eq. (15) in the Methods section, yields the ratio between the multipole relaxation times *T*_*P*_ = 3*T*_*d*_ = 6*T*_*f*_. Remarkably, the relaxation rates +3/2 ↔ + 1/2 and −3/2 ↔ −1/2 are not equal to that of +1/2 ↔ −1/2, and we obtain for them $(1/2)T_d^{ - 1}$ and $(2/3)T_d^{ - 1}$, respectively.

The analytical solution of the rate equations, Eq. (12) in Methods, shows that the strength of the modes (*s*, *s*′) = (+1, +1), (+1,−1), (−1,+1), and (−1,−1) are proportional to 5*T*_*d*_ − *T*_*p*_ − 4*T*_*f*_. Therefore, they vanish if the aforementioned ratio *T*_*p*_ = 3*T*_*d*_ = 6*T*_*f*_ holds. As a consequence, only four out of eight possible qudit modes are observed in Fig. 2a. The evolution of the qudit modes with *B*_*z*_, calculated as described in the Methods section, is shown in Fig. 2b. Here, we assume the Gaussian distribution of the zero-field splitting, $f(D) \propto exp[(D - \bar D)^2/(\delta D^2)]$, with the mean value $2\bar D = 26.8\,{\mathrm{MHz}}$ corresponding to the V3(V_Si_) center in 6H-SiC. A perfect agreement with the experimental data is achieved for $B_ \bot = 60\,\mu {\mathrm{T}}$, accounting for a uncompensated perpendicular component of the external magnetic field.

Some discrepancy between the measured and calculated images may stem from more general form of spin relaxation, not described by a single parameter, or more general form of inhomogeneous broadening.

### Coherent control of spin qudit modes

**Fig. 3:**
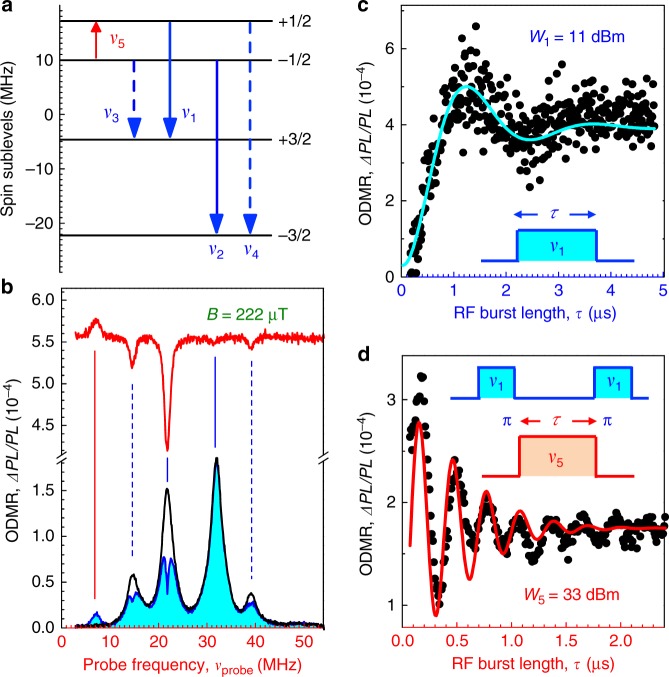
Coherent manipulation of spin qudit modes. **a** MW-induced transitions between different spin sublevels after optical pumping into the *m*_*S*_=±1/2 states. The solid and dashed arrows correspond to the transitions with large and small matrix elements, respectively. **b** ODMR spectra in a magnetic field of 222 μT with and without MW pump at *v*_pump_ = 21.8 MHz. The upper curve shows the spectrum of the excited qudit modes. **c** Rabi oscillations at the *v*_1_ resonance driven by the MW power *W*_1_ = 11 dBm with the corresponding *π*-pulse duration of 1.2 μs. **d** Rabi oscillations of a spin packet at the *v*_5_ resonance driven by the MW power *W*_5_ = 33 dBm

To further confirm the conclusions of our model, we investigate the qudit modes in a stronger magnetic field *B*_*z*_ = 211 μT when the Zeeman splitting is larger than inhomogeneous broadening. A precise analysis of the ODMR spectra^29,30^ shows that $B_ \bot = 73\,{\mathrm{\mu T}}$ remains almost the same. Figure 3a shows relative positions of the V_Si_ spin sublevels calculated for this field configuration. The ODMR spectrum consists of four lines [the lower curve in Fig. 3b] and the corresponding spin transitions enumerated from *v*_1_ to *v*_4_ are shown by arrows in Fig. 3a. The inner transitions *v*_1,2_ are stronger than the outer transitions *v*_3,4_, in accordance with earlier studies^28^.

Now, we burn a spectrally narrow hole at *v*_1_ (*v*_pump_ = 21.8 MHz), as shown in Fig. 3b. As expected from our model, excitation of qudit modes leads to the emergence of spectral holes at the *v*_2_, *v*_3_, and *v*_4_ transitions [see the upper curve in Fig. 3b]. Their spectral positions shift linearly with *v*_pump_, keeping the frequency difference the same. This is particularly pronounced in another sample with much larger inhomogeneous broadening, isotopically purified 4H-^28^SiC (Supplementary Fig. 5). We hence conclude that by varying *v*_pump_ within the *v*_1_ ODMR line, different spin packets are selected. Remarkably, there is another signal at *v*_5_ in Fig. 3b, which has the opposite sign and appears only if the *v*_1_ qudit mode is excited. The same behavior is also observed in 4H-SiC^30^ (see also Supplementary Fig. 6). These general properties are used to implement spectrally selective coherent control of qudit modes.

We start with driving the *v*_1_ transition. Figure 3c shows Rabi oscillations when the driving power is relatively low *W*_1_ = 11 dBm. The corresponding *π*-pulse duration of 1.2 μs provides sub-MHz spectral selectivity. We then spectrally select a spin packet using a long *π* pulse at *v*_1_ and coherently drive it at the *v*_5_ resonance followed by a second long *π* pulse at *v*_1_ for the readout [inset of Fig. 3d]. A high driving power *W*_5_ = 33 dBm yields fast Rabi oscillations, as presented in Fig. 3d. For such a power, the *π*/2 pulse is 80 ns, corresponding to a bandwidth of ~10 MHz. This bandwidth is wide enough to encompass the *v*_5_ linewidth.

### Two-frequency Ramsey interferometry

**Fig. 4:**
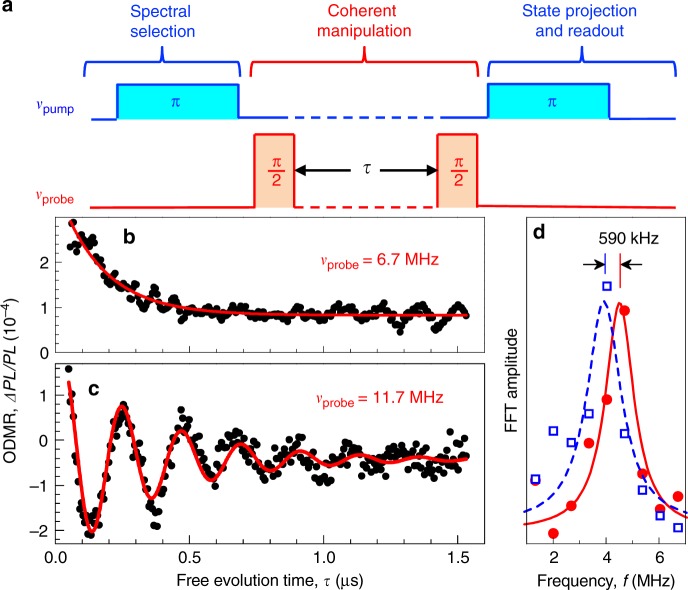
Ramsey interferometry of spin qudit modes. **a** Pulse pattern for the spectral selection, coherent manipulation, and state projection followed by the readout of spin packets. **b** Ramsey measurement of the spin packet selected by *v*_pump_ = 21.8 MHz in a magnetic field of 223 ± 2 μT. The probe frequency *v*_probe_ = 6.7 MHz is set to the *v*_5_ resonance. The solid line represents the fit to an exponential decay with $T_2^ \ast = 168 \pm 7\,{\mathrm{ns}}$. **c** The same as (**b**) but the probe frequency *v*_probe_ = 11.7 MHz is detuned from the *v*_5_ resonance. The solid line represents the fit to an exponentially decaying sinusoid with $T_2^ \ast = 357 \pm 24\,{\mathrm{ns}}$. **d** FFT of the Ramsey fringes fitted to a Lorentz function. The solid line corresponds to the data from (**c**). The dashed line is for the same spin packet as in **c** but probed in a magnetic field of 242 ± 3 μT

To highlight the advantages of qudit modes for quantum information processing and sensing, we demonstrate Ramsey-based absolute magnetometry performing two-frequency experiments with the protocol shown in Fig. 4a (and Supplementary Fig. 2). When the probe frequency *v*_probe_ = 6.7 MHz is equal to the *v*_5_ qudit mode frequency, the signal represents free induction decay (Fig. 4b). We note that though the other, *v*_3_ resonance lies within the bandwidth, the population of the *m*_*S*_ = +3/2 and *m*_*S*_ = −1/2 is equal after the *v*_1_ *π*-pump pulse (Fig. 3a), and this resonance is not driven. This is also confirmed by the absence of pronounced fringes in the free induction decay of Fig. 4b.

When *v*_probe_ = 11.7 MHz is detuned from the *v*_5_ mode, Ramsey fringes are clearly detected (Fig. 4c). A fit of these dynamics to $cos(2\pi f_R\tau )exp( - \tau /T_2^ \ast )$ gives the coherence time of spin packets $T_2^ \ast = 357 \pm 24\,{\mathrm{ns}}$. For comparison, we also perform standard Ramsey measurements using single MW frequency, but do not observed fringes because of inhomogeneous broadening and non-selective excitation (Supplementary Fig. 7).

Solid circles in Fig. 4d represent the fast Fourier transform (FFT) of the experimental data from Fig. 4c. The fitting of these data to a Lorentz function yields the frequency of the Ramsey fringes *fR* = 4.51 ± 0.03 MHz, with the corresponding spectral resolution of 30 KHz. Following Eq. (1), the effective magnetic field can be measured with high accuracy as

3 $$B_{{\mathrm{eff}}} = \frac{{\nu _{{\mathrm{probe}}} - f_{\mathrm{R}}}}{{\gamma \sqrt {1 + 3\mathop {{sin}}\nolimits^2 (\theta )} }}.$$

Here, *θ* is the angle between the magnetic field direction and the *c*-axis of SiC. It is calculated from the ratio (*v*_2_ − *v*_1_)/(*v*_4_− *v*_3_) with resolution better than 1° following the previously described procedure^29^, which yields *θ* = 19° ± 0.5°. The gyromagnetic ratio *γ* is the constant value. We then obtain from Eq. (3) *B*_eff_ = 223 ± 2 μT. To analyze the spectral selectivity of different spin packets, we marginally vary the external magnetitic field strength and the pump/probe frequencies. The results are summarized in Table 2, where measurement No. 1 corresponds to the case discussed above. We then increase *B* and change *v*_pump_ to select the same spin packet (measurement No. 2 in Table 2). The corresponding FFT of the Ramsey fringes is represented by open squares in Fig. 4d and a lorentzian fit (the dashed line) yields *f*_*R*_ = 3.92 ± 0.05 MHz and *B*_eff_ = 242 ± 3 μT. A comparison of measurements No. 1 and No. 2 demonstrates the spectral selectivity of spin packets to be ~600 kHz. We note that an unusually narrow ODMR linewidth of 500 kHz in SiC was observed earlier in two-frequency experiments, but its origin had not been discussed^30^.

**Table 2:** FFT frequency of the Ramsey fringes *f*_R_ in 6H-^28^SiC for different pump *v*_pump_ and probe *v*_probe_ frequencies in different magnetic fields *B* (bias coil currents *I*)

No.	*I* (mA)	*v*_pump_ (MHz)	*v*_probe_ (MHz)	*f*_R_ (MHz)	*B*_eff_ (μT)
1	100	21.80	11.70	4.51 ± 0.03	223 ± 2
2	105	21.20	11.70	3.92 ± 0.05	242 ± 3
3	100	21.20	11.70	4.51 ± 0.04	223 ± 2

Experimental data are presented in Fig. 4 and Supplementary Fig. 8

## Discussion

Measurements Nos. 1 3 in Table 2 demonstrate that the effective magnetic field *B*_eff_ seen by different spin packets, which are selected by different *v*_pump_, is the same within the error bars. This is a manifestation that the inhomogeneous broadening is caused mostly by local variations of the zero-field splitting 2*D* rather than magnetic fluctuations. Remarkably, another pair of measurements (No. 2 and No. 3) in Table 2 show that the magnetic field strength can be measured without calibration of the zero-field splitting, which can be used to implement absolute (i.e., immune to thermal noise and strain inhomogeneity) DC magnetometry^31^.

To summarize, we demonstrated coherent manipulation of spin qudit modes in isotopically purified SiC at room temperature. We also developed a theory describing the excitation and detection of these modes in inhomgeneously broadened systems and showed that qudits are characterized by multiple relaxation times. These findings can lead to dipole-coupled networks^11^, unconditional electron-nuclear spin registers^14^, and spectral selection of highly coherent individual spins^15,22,32^, particularly in nanocrystals^33^. Our results hence open new possibilities to improve the sensitivity of quantum sensors and execute nontrivial quantum protocols in dense spin ensembles.

## Methods

### Samples preparation

The semi-insulating (SI) 4H-SiC substrate with natural isotope content (4.7% of ^29^Si and 1.1% of ^13^C) was purchased from Cree. The 6H- and 4H-^28^SiC samples, purified from the ^29^Si isotope, were grown by the seeded physical vapour transport method^34^ with a ^28^Si enriched precursor material on a 6H-SiC substrate with natural isotope content. The ^29^Si isotope abundance in these samples of 1% was established by the EPR spectroscopy (Supplementary Note and Supplementary Fig. 1). To create V_Si_ centers, the 6H-^28^SiC and SI 4H-SiC samples were irradiated with 2 MeV electrons at temperatures close to room temperature to a total dose of 10^18^ cm^−2^. To create V_Si_ centers in 4H-^28^SiC, the sample was irradiated with 3–5 MeV neutrons at temperatures close to room temperature to a total dose of 10^15^ cm^−2^.

### Two-frequency optically detected magnetic resonance (ODMR)

A scheme of the experimental setup used in our ODMR/hole-burning experiments is presented in Supplementary Fig. 2. A 787.7 -nm diode laser (LD785-SE400 from Thorlabs) was pulsed using an acousto-optic modulator (AOM A&A Opto-Electronic MT250-A02-800). The free space laser beam was coupled by several mirrors into a 50-μm optical fiber, which transfers the light through a ×10 microscope objective (Olympus LMPLN10XIR). The objective focused the beam on the sample surface with a spot diameter of approximately 10 μm. The laser power was about 10 mW at the sample surface. The PL was collected by the same ×10 objective and filtered by an 850 -nm and 875 -nm long pass before it was coupled into a 600 -nm optical fiber. As a detector, a Si avalanche photodiode was used (APD120A from Thorlabs). The signal was processed by a lock-in amplifier (Signal Recovery DSP 7230). Radiofrequency was generated by a signal generator (Stanford Research Systems SG384) and amplified by an amplifier (Vectawave VBA1000-18) before being supplied to the sample by a 0.5 -mm copper-stripline the sample was placed on. In the hole burning experiments, the second radiofrequency was supplied by an identical Stanford Research Systems SG384 generator. The radio waves were modulated using RF-switches (Mini-Circuits ZASWA-2-50DR+) and a mixed before entering the amplifier using combiner (Mini-Circuits ZFSC-2-4-S+). To apply the external magnetic field, a permanent magnet below the sample and Helmholtz coil was used.

### Multipole decomposition

An ensemble of spin-3/2 centers is described by the 4 × 4 spin-density matrix *ρ*, which can be decomposed into a sum of the 16 basis matrices,

4 $$\rho = \mathop {\sum}\limits_{i = 1}^{16} {{\mathrm{Tr}}} ({\cal{M}}_i^\dagger \rho )\;{\cal{M}}_i.$$

The matrices ${\cal{M}}_j$ comprise three matrices of the spin dipole,

5 $$\begin{array}{l}{\hskip-2.7pc}{\cal{P}}_0 = \left( {\begin{array}{*{20}{c}} {\frac{3}{{2\sqrt 5 }}} & 0 & 0 & 0 \\ 0 & {\frac{1}{{2\sqrt 5 }}} & 0 & 0 \\ 0 & 0 & { - \frac{1}{{2\sqrt 5 }}} & 0 \\ 0 & 0 & 0 & { - \frac{3}{{2\sqrt 5 }}} \end{array}} \right),\\ {\cal{P}}_{ + 1} = \left( {\begin{array}{*{20}{c}} 0 & {\sqrt {\frac{3}{{10}}} } & 0 & 0 \\ 0 & 0 & {\sqrt {\frac{2}{5}} } & 0 \\ 0 & 0 & 0 & {\sqrt {\frac{3}{{10}}} } \\ 0 & 0 & 0 & 0 \end{array}} \right),{\cal{P}}_{ - 1} = {\cal{P}}_{ + 1}^\dagger ,\end{array}$$

five matrices of the spin quadrupole,

6 $$\begin{array}{l}{\cal{D}}_0 = \left( {\begin{array}{*{20}{c}} {\frac{1}{2}} & 0 & 0 & 0 \\ 0 & { - \frac{1}{2}} & 0 & 0 \\ 0 & 0 & { - \frac{1}{2}} & 0 \\ 0 & 0 & 0 & {\frac{1}{2}} \end{array}} \right),{\cal{D}}_{ + 1} = \left( {\begin{array}{*{20}{c}} 0 & { - \frac{1}{{\sqrt 2 }}} & 0 & 0 \\ 0 & 0 & 0 & 0 \\ 0 & 0 & 0 & {\frac{1}{{\sqrt 2 }}} \\ 0 & 0 & 0 & 0 \end{array}} \right),\\ {\hskip-2.3pc}{\cal{D}}_{ + 2} = \left( {\begin{array}{*{20}{c}} 0 & 0 & {\frac{1}{{\sqrt 2 }}} & 0 \\ 0 & 0 & 0 & {\frac{1}{{\sqrt 2 }}} \\ 0 & 0 & 0 & 0 \\ 0 & 0 & 0 & 0 \end{array}} \right),{\cal{D}}_{ - 1} = {\cal{D}}_{ + 1}^\dagger ,\;{\cal{D}}_{ - 2} = {\cal{D}}_{ + 2}^\dagger ,\end{array}$$

seven matrices of the spin octupole,

7 $$\begin{array}{l}{\hskip-8.8pc}{\cal{F}}_0 = \left( {\begin{array}{*{20}{c}} {\frac{1}{{2\sqrt 5 }}} & 0 & 0 & 0 \\ 0 & { - \frac{3}{{2\sqrt 5 }}} & 0 & 0 \\ 0 & 0 & {\frac{3}{{2\sqrt 5 }}} & 0 \\ 0 & 0 & 0 & { - \frac{1}{{2\sqrt 5 }}} \end{array}} \right),\\ {\cal{F}}_{ + 1} = \left( {\begin{array}{*{20}{c}} 0 & {\frac{1}{{\sqrt 5 }}} & 0 & 0 \\ 0 & 0 & { - \sqrt {\frac{3}{5}} } & 0 \\ 0 & 0 & 0 & {\frac{1}{{\sqrt 5 }}} \\ 0 & 0 & 0 & 0 \end{array}} \right),\quad {\cal{F}}_{ + 2} = \left( {\begin{array}{*{20}{c}} 0 & 0 & {\frac{1}{{\sqrt 2 }}} & 0 \\ 0 & 0 & 0 & { - \frac{1}{{\sqrt 2 }}} \\ 0 & 0 & 0 & 0 \\ 0 & 0 & 0 & 0 \end{array}} \right),\\ {\hskip1.5pc}{\cal{F}}_{ + 3} = \left( {\begin{array}{*{20}{c}} 0 & 0 & 0 & { - 1} \\ 0 & 0 & 0 & 0 \\ 0 & 0 & 0 & 0 \\ 0 & 0 & 0 & 0 \end{array}} \right),\begin{array}{*{20}{l}} {{\cal{F}}_{ - 1} = {\cal{F}}_{ + 1}^\dagger ,\;{\cal{F}}_{ - 2} = {\cal{F}}_{ + 2}^\dagger ,\;{\cal{F}}_{ - 3} = {\cal{F}}_{ + 3}^\dagger ,} \hfill \end{array}\end{array}$$

and the diagonal matrix ${\cal{I}}/2$, with ${\cal{I}}$ being the unit matrix. The above matrices form a complete orthogonal basis, ${\mathrm{Tr}}({\cal{M}}_i^\dagger {\cal{M}}_j) = \delta _{ij}$.

### Calculation of ODMR spectra

The density matrix describing an ensemble of spin centers satisfies the equation^20^,

8 $$\frac{{d\rho }}{{dt}} = \frac{{\mathrm{i}}}{\hbar }[\rho ,H] + G + (\dot \rho )_{{\mathrm{rel}}},$$

where *H* is the Hamiltonian,

9 $$H = D\left( {S_z^2 - \frac{5}{4}} \right) + \hbar \gamma [{\boldsymbol{B}} + {\tilde{\boldsymbol{B}}}(t)] \cdot {\boldsymbol{S}},$$

$\widetilde {\boldsymbol{B}}(t) = \mathop {\sum}\nolimits_{\alpha = 1,2} {\left( {{\boldsymbol{B}}_\alpha {\mathrm{e}}^{ - {\mathrm{i}}\omega _\alpha t} + {\mathrm{c}}.c.} \right)}$ is the magnetic field of the two-frequency (pump and probe) MW radiation, and 2*D* is the zero-field splitting. Optical initialization of the spin centers is described by the term $G = \eta {\cal{D}}_0I_0$, where ${\cal{D}}_0$ is the spin quadrupole given by Eq. (6), *η* is a material parameter (*η* > 0 or *η* < 0 depending on whether the *m*_*S*_ = ±3/2 or *m*_*S*_ = ±1/2 spin states are preferentially populated at optical excitation), and *I*_0_ is the light intensity.

Spin relaxation is taken into account in Eq. (8) by the term

10 $$\begin{array}{*{20}{l}} {(\dot \rho )_{{\mathrm{rel}}} = - \frac{{\rho _{\cal{P}}}}{{T_p}} - \frac{{\rho _{\cal{D}}}}{{T_d}} - \frac{{\rho _{\cal{F}}}}{{T_f}},} \hfill \end{array}$$

where $\rho _{\cal{P}} = \mathop {\sum}\nolimits_{k = - 1}^1 {{\mathrm{Tr}}} ({\cal{P}}_k^\dagger \rho ){\cal{P}}_k$, $\rho _{\cal{D}} = \mathop {\sum}\nolimits_{k = - 2}^2 {{\mathrm{Tr}}} ({\cal{D}}_k^\dagger \rho ){\cal{D}}_k$, and $\rho _{\cal{F}} = \mathop {\sum}\nolimits_{k = - 3}^3 {{\mathrm{Tr}}} ({\cal{F}}_k^\dagger \rho ){\cal{F}}_k$ are the dipole, quadrupole, and octupole contributions to the density matrix, respectively, *T*_*p*_, *T*_*d*_, and *T*_*f*_ are the corresponding relaxation times.

The spin-density matrix *ρ* found from Eq. (8) is used to calculate the spin-dependent correction to the PL intensity^20^

1 $$\Delta {\mathrm{PL}}/{\mathrm{PL}} = \alpha {\mathrm{Tr}}({\cal{D}}_0\rho ){\kern 1pt} ,$$

where *α* is a material constant. The result is then averaged over the ensemble of centers with slightly different values of the zero-filed splitting *D*, as described above.

### Semi-classical rate equations

Equation (8) is simplified in the basis of the eigenstates of the Hamiltonian (9) without the MW field. Disregarding the off-diagonal components of the density matrix, we obtain the semi-classical rate equations for the populations *ρ*_*ii*_ of the spin sublevels

12 $$\begin{array}{*{20}{l}} {\frac{{d\rho _{ii}}}{{dt}} = \mathop {\sum}\limits_j {W_{ij}} (\rho _{ii} - \rho _{jj}) + G_i - \mathop {\sum}\limits_j {R_{ij}} \rho _{jj}.} \hfill \end{array}$$

Here, *W*_*ij*_ are the rates of spin transitions by the MW fields,

13 $$\begin{array}{*{20}{l}} {W_{ij} = \mathop {\sum}\limits_\alpha {\frac{{2{\kern 1pt} \delta \omega _\alpha \hbar ^2\gamma ^2\left| {{\boldsymbol{B}}_\alpha \cdot {\boldsymbol{S}}_{ij}} \right|^2}}{{(|E_i - E_j| - \hbar \omega _\alpha )^2 + (\hbar {\kern 1pt} \delta \omega _\alpha )^2}}} {\kern 1pt} ,} \hfill \end{array}$$

*δω*_*α*_ are the halfwidths of the MW field spectra, which are supposed to have the Lorentzian shape, $G_i = \eta \left[ {{\cal{D}}_0} \right]_{ii}I_0$, and *R*_*ij*_ = *R*_*ji*_ are the component of the matrix of relaxation rates

14 $$\begin{array}{*{20}{l}} {R_{ij}} \hfill & = \hfill & {\frac{1}{{T_p}}\mathop {\sum}\limits_{k = - 1}^1 {\left[ {{\cal{P}}_k} \right]_{ii}} \left[ {{\cal{P}}_k} \right]_{jj}^ \ast + \frac{1}{{T_d}}\mathop {\sum}\limits_{k = - 2}^2 {\left[ {{\cal{D}}_k} \right]_{ii}} \left[ {{\cal{D}}_k} \right]_{jj}^ \ast } \hfill \\ {} \hfill & {} \hfill & { + \frac{1}{{T_f}}\mathop {\sum}\limits_{k = - 3}^3 {\left[ {{\cal{F}}_k} \right]_{ii}} \left[ {{\cal{F}}_k} \right]_{jj}^ \ast .} \hfill \end{array}$$

For ***B***||*z*, the components *R*_*ij*_ have a particular simple form

15 $$\begin{array}{*{20}{l}} {R_{ + 3/2, + 3/2}} \hfill & = \hfill & {R_{ - 3/2, - 3/2} = \frac{1}{{20}}\left( {\frac{9}{{T_p}} + \frac{5}{{T_d}} + \frac{1}{{T_f}}} \right){\kern 1pt} ,} \hfill \\ {R_{ + 3/2, + 1/2}} \hfill & = \hfill & {R_{ - 3/2, - 1/2} = \frac{1}{{20}}\left( {\frac{3}{{T_p}} - \frac{5}{{T_d}} - \frac{3}{{T_f}}} \right){\kern 1pt} ,} \hfill \\ {R_{ + 3/2, - 1/2}} \hfill & = \hfill & {R_{ - 3/2, + 1/2} = \frac{1}{{20}}\left( { - \frac{3}{{T_p}} - \frac{5}{{T_d}} + \frac{3}{{T_f}}} \right){\kern 1pt} ,} \hfill \\ {R_{ + 3/2, - 3/2}} \hfill & = \hfill & {\frac{1}{{20}}\left( { - \frac{9}{{T_p}} + \frac{5}{{T_d}} - \frac{1}{{T_f}}} \right){\kern 1pt} ,} \hfill \\ {R_{ + 1/2, + 1/2}} \hfill & = \hfill & {R_{ - 1/2, - 1/2} = \frac{1}{{20}}\left( {\frac{1}{{T_p}} + \frac{5}{{T_d}} + \frac{9}{{T_f}}} \right){\kern 1pt} ,} \hfill \\ {R_{ + 1/2, - 1/2}} \hfill & = \hfill & {\frac{1}{{20}}\left( { - \frac{1}{{T_p}} + \frac{5}{{T_d}} - \frac{9}{{T_f}}} \right){\kern 1pt} .} \hfill \end{array}$$

The PL correction is given by ${\mathrm{\Delta PL}}/{\mathrm{PL}} = \alpha \mathop {\sum}\nolimits_i \left[ {{\cal{D}}_0} \right]_{ii}\rho _{ii}$.

## Supplementary information

Supplementary Information

Peer Review

## Data Availability

The data supporting this study are available from the corresponding author upon reasonable request.
