# Supplementary material for: Excitation and coherent control of spin qudit modes in silicon carbide at room temperature
Source: Nat Commun. 2019 Apr 11;10:1678. doi: 10.1038/s41467-019-09429-x (PMC6459825; doi:10.1038/s41467-019-09429-x)
Supplement: Supplementary file 1 — Supplementary Information [file 41467_2019_9429_MOESM1_ESM.pdf]

Supplementary Information

Excitation and coherent control of spin qudit modes in silicon carbide at room temperature

Soltamov et al.

SUPPLEMENTARY NOTE: INVESTIGATED SAMPLES

Silicon carbide crystals 6H-^{28}SiC purified from the ^{29}Si isotope were grown by the seeded physical vapour transport method [1, 2] with a ^{28}Si enriched precursor material. As a seed a 6H-SiC substrate with natural isotope content was used ^{29}Si ($I = 1/2$, 4.7% natural abundance) and 13C ($I = 1/2$, 1.1% natural abundance). To estimate the content of the ^{29}Si isotope in the grown sample, X-band ($\nu = 9.4$ GHz) Electron Paramagnetic Resonance (EPR) spectroscopy was used.

The EPR spectrum of the sample recorded in the magnetic field oriented parallel to the c-axis at a temperature 150 K under continuous light illumination ($\lambda = 808$ nm) is presented in Supplementary Figure 1 (a). Two pairs of fine-structure EPR transitions are observed with the zero field splitting parameter $2D$ of 27 MHz and 128 MHz. These EPR lines are the signatures of the negatively-charged silicon vacancies in 6H-SiC [3]. Each fine structure line (FS) corresponding to the transitions with $I = 0$ is accompanied by the doublet of hyperfine lines (HF) with 0.29 mT splitting arising due to the ligand HF interaction with one ^{29}Si atom ($I = 1/2$) among the 12 next-nearest neighbors Si atoms [3–5] (see Supplementary Figure 1 (b)). The ratio of the intensity between the total intensity of the two HF lines and the FS line is calculated to be $I_{HF}/I_{FS} = 0.126$. We than use this ratio to determine the ^{29}Si concentration in our 6H-^{28}SiC sample, taking into account that the HF line intensity is determined by the probability to find one ^{29}Si atom among the 12 next-nearest neighbors Si atoms (P_1), while the intensity of the FS line is determined by the probability that all 12 Si atoms have $I = 0$ (P_0). The probability is determined by the Eq. (S1) [6]

$$P_m = C_n^m X^m (1 - X)^{n-m}, \tag{S1}$$

where P_m is the probability for a lattice site to be occupied by an odd isotope, m is the number of sites occupied by the isotope, n is the number of the equivalent lattice sites under consideration and X is the isotope concentration (^{29}Si). Following the Eq. (S1) $P_1 = 12 \times X \times (1 - X)^{11}$, $P_0 = (1 - X)^{12}$. Using the ratio $I_{HF}/I_{FS} = 0.126$, the $X = 0.0103$ is obtained. Thus, the ^{29}Si content in the sample is approximately 1%. The simulation of the EPR lineshape made in the Bruker BioSpin software taking in consideration the Si content to be 1% is shown in Fig. 1(b). The good agreement of the experimental spectrum and the simulated one is seen that supports our calculations.

SUPPLEMENTARY REFERENCES

[1] Heydemann, V. D., Schulze, N., Barrett, D. L. & Pensl, G. Growth of 6h and 4h silicon carbide single crystals by the modified lely process utilizing a dual-seed crystal method. *Applied Physics Letters* **69**, 3728–3730 (1996).

[2] Mokhov, E., Ramm, M., Roenkov, A. & Vodakov, Y. Growth of silicon carbide bulk crystals by the sublimation sandwich method. *Materials Science and Engineering: B* **46**, 317 – 323 (1997).

[3] von Bardeleben, H. J., Cantin, J. L., Vickridge, I. & Battistig, G. Proton-implantation-induced defects in n-type $6h$- and $4h-$SiC : an electron paramagnetic resonance study. *Phys. Rev. B* **62**, 10126–10134 (2000).

[4] Wimbauer, T., Meyer, B. K., Hofstaetter, A., Scharmann, A. & Overhof, H. Negatively charged si vacancy in $4h$ sic: A comparison between theory and experiment. *Phys. Rev. B* **56**, 7384–7388 (1997).

[5] Mizuochi, N. *et al.* Spin multiplicity and charge state of a silicon vacancy (T_{V2a}) in $4h$-sic determined by pulsed endor. *Phys. Rev. B* **72**, 235208 (2005).

[6] Hughes A. E., H. B. *Color centers in Simple Oxides. In: Point Defects in Solids. Ed. by Crawford J. H., Slifkin L. W.* (Springer, Boston, MA, 1972).

SUPPLEMENTARY FIGURES

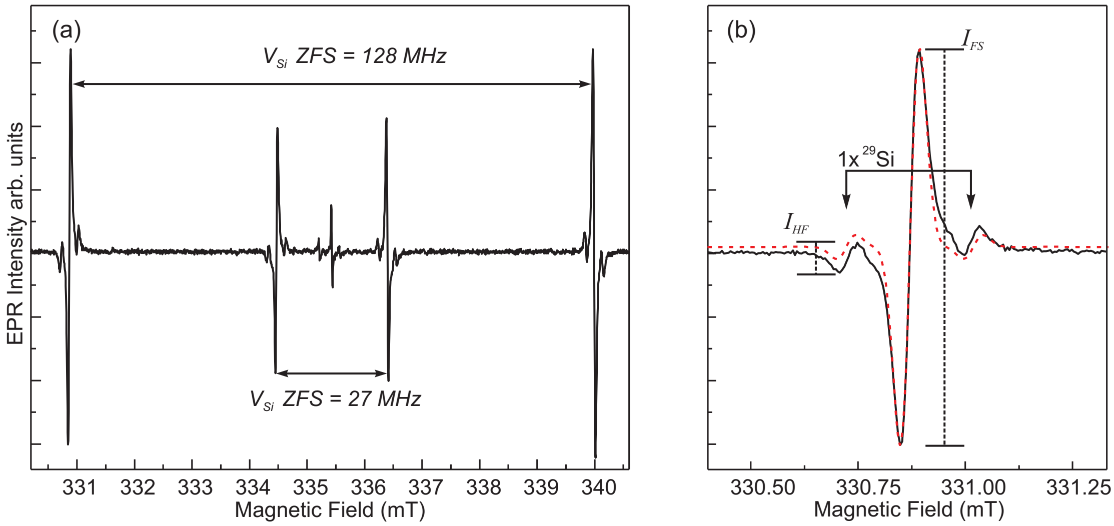

Supplementary Figure 1: (a) EPR spectrum of the 6H-^{28}SiC samples. EPR signals of the V_{Si} centers are indicated by arrows. (b) Low magnetic field EPR line of the V_{Si} shown in the enlarged scale. Vertical bars indicate pick-to-pick intensities of the HF and FS lines. Vertical arrows indicate the doublet of hyperfine lines with 0.29 mT splitting arising due to the HF interaction with one ^{29}Si atom among the 12 next-nearest neighbors Si atoms. Experimental spectrum is shown with the black solid line, the simulated EPR line assuming ^{29}Si concentration to be 1%, is shown with the dashed red line.

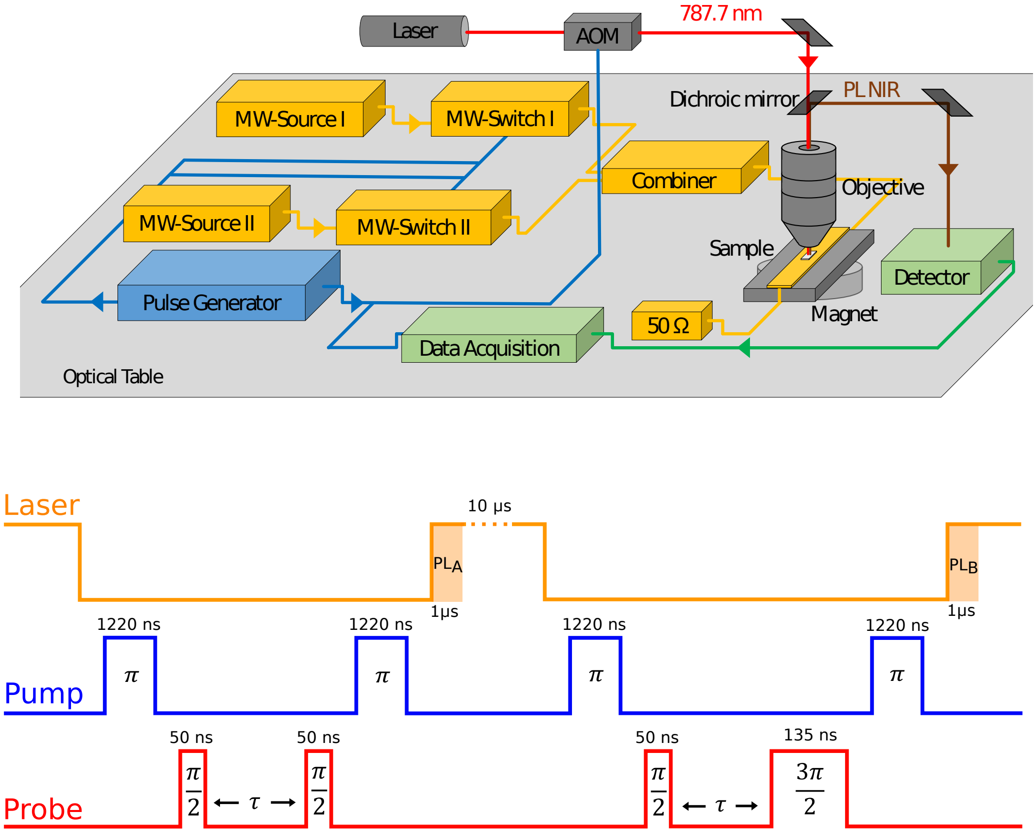

Supplementary Figure 2: Top: A scheme of the experimental setup. Bottom: A pulse sequence to perform two-frequency Ramsey measurements. The ODMR signal is obtained as $\mathrm{PL_A} - \mathrm{PL_B}/\mathrm{PL}$. Here, $\mathrm{PL_A}$ and $\mathrm{PL_B}$ are recorded after MW probe sequences $\pi/2 - \tau - \pi/2$ and $\pi/2 - \tau - 3\pi/2$, respectively.

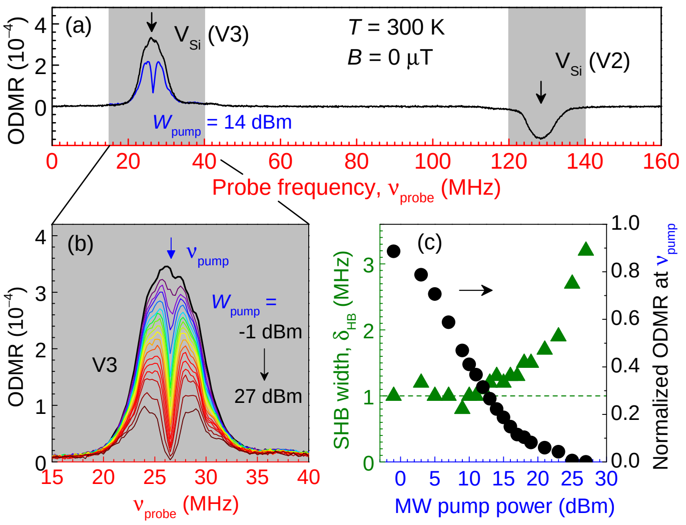

Supplementary Figure 3: Two-frequency ODMR spectroscopy. (a) ODMR spectrum of isotopically purified 6H-SiC without and with the second pump MW field at $\nu_{\mathrm{pump}} = 26.8\,\mathrm{MHz}$. The pump and probe powers are $W_{\mathrm{probe}} = 7\,\mathrm{dBm}$ and $W_{\mathrm{pump}} = 14\,\mathrm{dBm}$, correspondingly. (b) SHB at the V3 spin resonance for different W_{pump}. (c) Pump power dependence of the SHB width δ_{HB} and the normalized ODMR signal at $\nu_{\mathrm{pump}} = 26.8\,\mathrm{MHz}$. The horizontal dashed line indicates the limit of δ_{HB} for low W_{pump}.

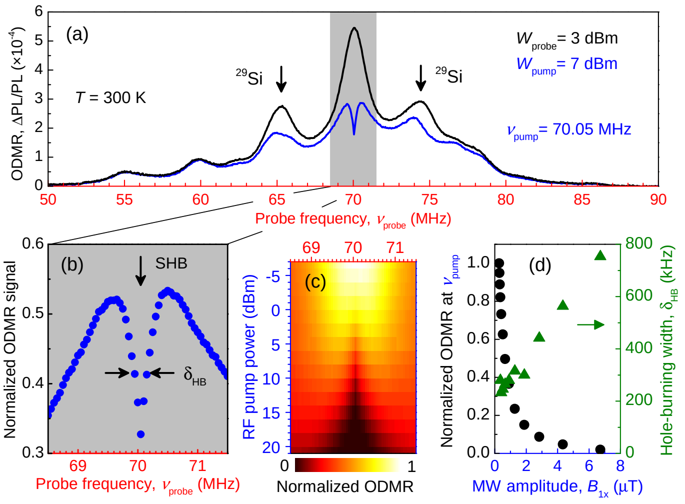

Supplementary Figure 4: (a) ODMR spectrum of 4H-SiC without and with the second pump MW field in the earth magnetic field. (b) Zoom-in into the spin resonance at 70 MHz associated with the V2 silicon vacancy. (c) Power dependence of SHB. (d) SHB deep and width as a function of MW field amplitude.

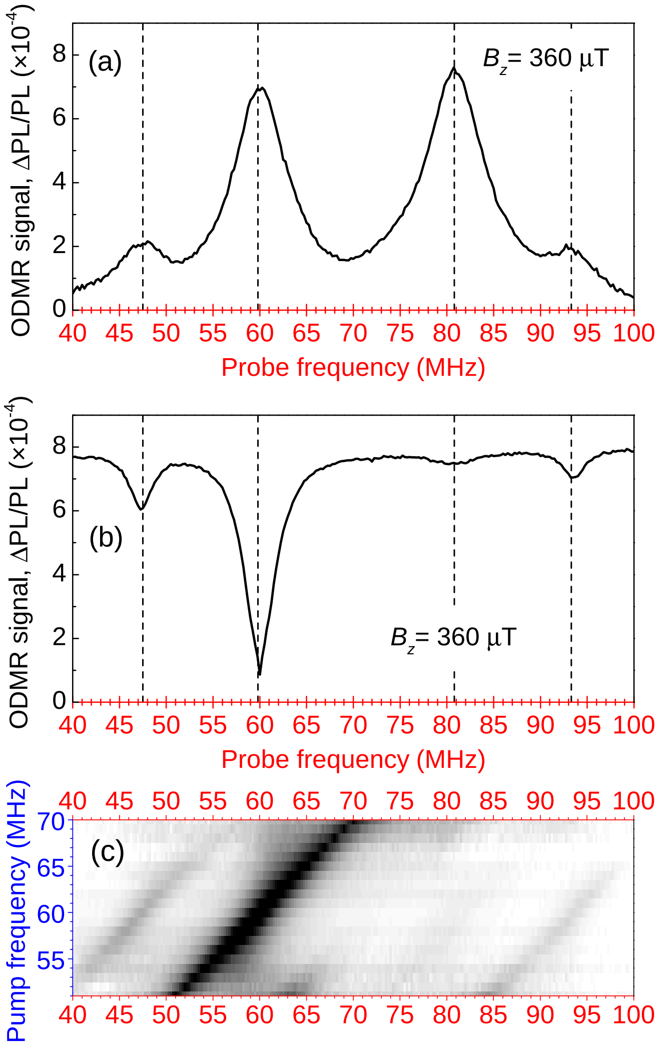

Supplementary Figure 5: (a) ODMR spectrum of 4H-^{28}SiC in a magnetic field $B_z = 360\,\mu$T (perpendicular component of the magnetic field is not compensated). (b) Pump-induced ($\nu_{\rm pump} = 60\,$MHz) changes in the ODMR spectrum. (c) Pump-probe frequency scans showing relative shifts of the SHB satellites.

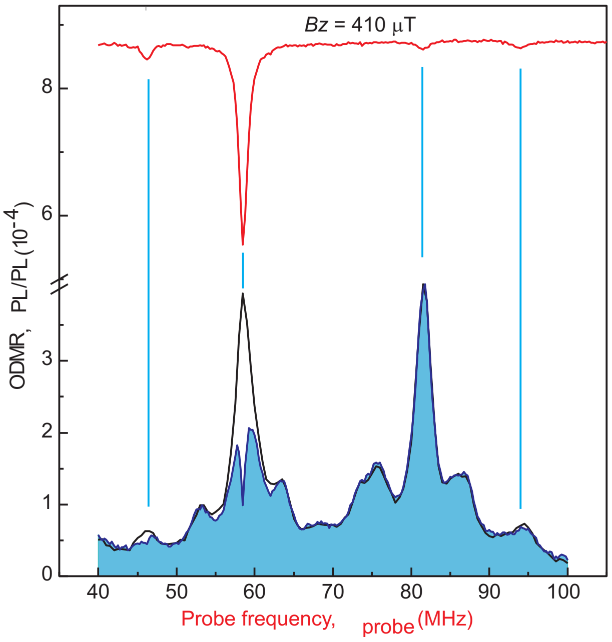

Supplementary Figure 6: ODMR spectra of the V_{Si} centers in 4H-SiC detected in the external magnetic field $B_z = 410\,\mu T$ applied parallel to the c-axis (perpendicular component due to the earth magnetic field is not compensated) with and without SHB at $\nu_{pump} = 58.5\,MHz$. The upper curve is obtained under pump modulation.

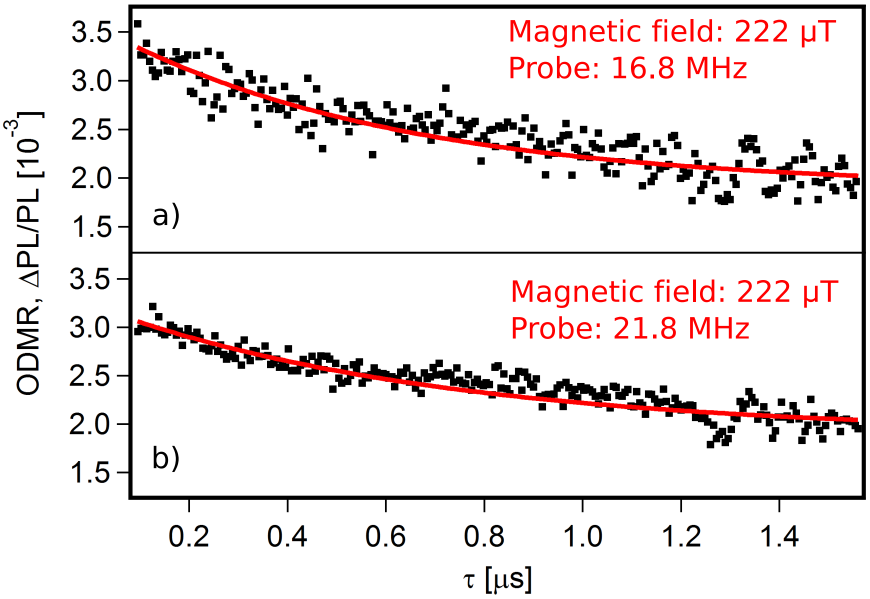

Supplementary Figure 7: Single-frequency Ramsey measurements in isotopically purified 6H-^{28}SiC. (a) Ramsey measurement at the ν_1 resonance. (b) Ramsey measurement with MW frequency detuned from the ν_1 resonance.

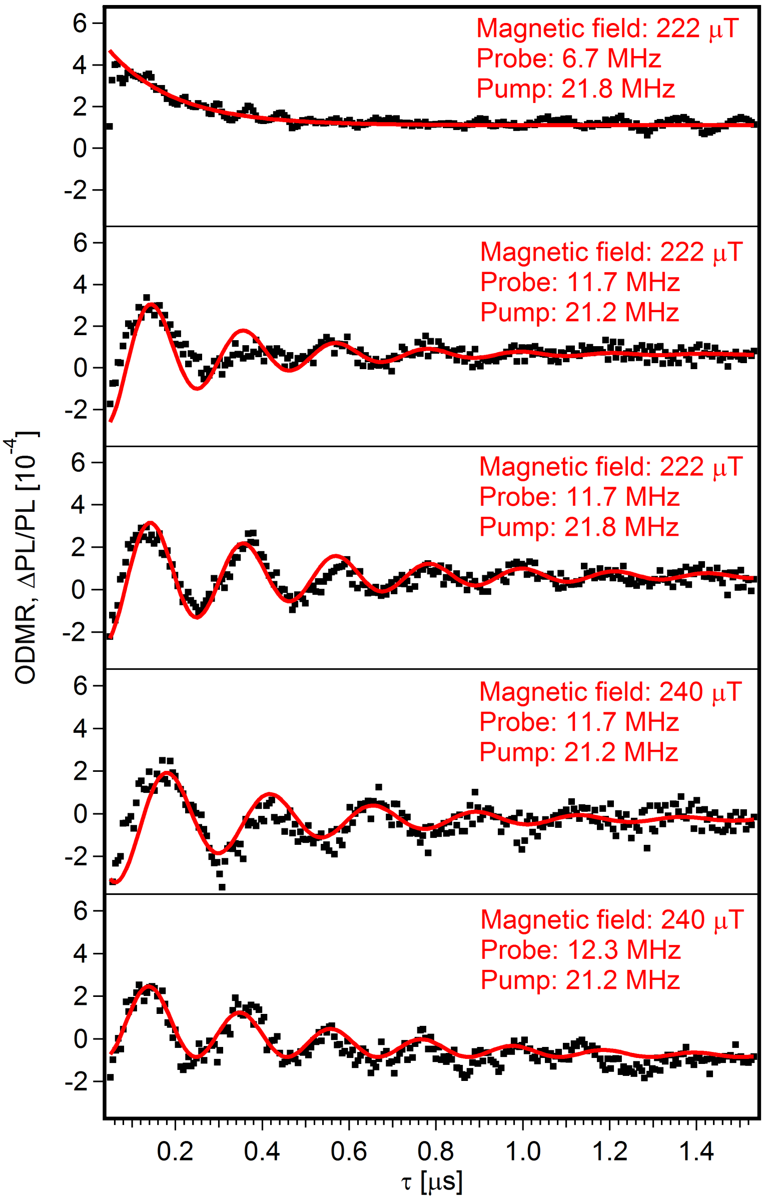

Supplementary Figure 8: Ramsey measurements of spectrally selected spin packets in isotopically purified 6H-^{28}SiC. The frequencies of the ν_5 and ν_1 resonance at 222 μT are 6.7 MHz and 21.8 MHz, correspondingly. At 240 μT the ν_5 resonance is at 7.3 MHz and ν_1 resonance is 21.2 MHz.

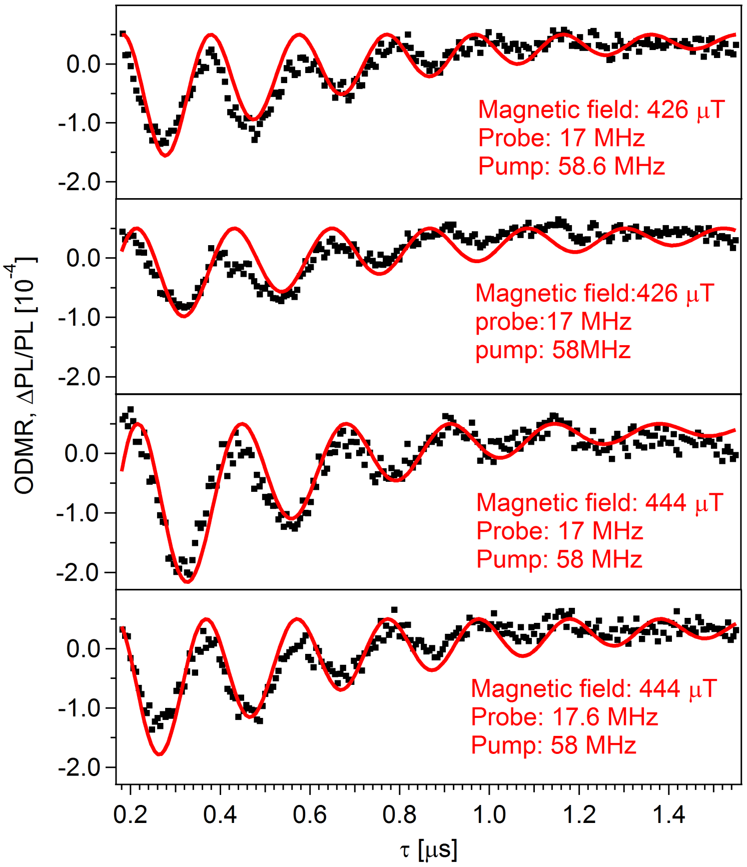

Supplementary Figure 9: Ramsey measurements of spectrally selected spin packets in 4H-SiC. The frequency of the ν_5 resonance is at 12 MHz for 426 μT and 12.6 MHz for 426 μT . The frequency of the ν_1 resonance is at 58.6 MHz and 58 MHz for 426 μT and 444 μT, correspondingly. The solid lines represent the fit to an exponentially decaying sinusoid.
