# Supplementary material for: Excitation and coherent control of spin qudit modes in silicon carbide at room temperature
Source: Nat Commun. 2019 Apr 11;10:1678. doi: 10.1038/s41467-019-09429-x (PMC6459825; doi:10.1038/s41467-019-09429-x)
Supplement: Supplementary file 2 — Peer Review [file 41467_2019_9429_MOESM2_ESM.pdf]

Reviewers' comments:

Reviewer #1 (Remarks to the Author):

The manuscript "Excitation and coherent control of spin qudit modes with sub-MHz spectral resolution" by V.A. Soltamov et al. deals with the excitation and control of spin qudits based on a silicon carbide platform at room temperature. The background of different polymorphs and the related theory are first discussed, followed by the characterization of the sample by means of optically detected magnetic resonance and electron paramagnetic resonance. Prior to the experimental and numerical part, different broadening mechanisms are examined in order to give a better understanding of the spin qudit spectrum. Finally, simulations and experiments are performed, which demonstrate the excitation of different spin qudit modes, as well as their coherent control by means of Ramsey interferometry.

The submitted manuscript demonstrates new schemes for the coherent control of solid state spin qudits at room temperature, which represents a novelty in the solid-state field. However, before considering acceptance in Nature Communications, I would suggest the following major revisions.

On the first page, the authors claim that the 29Si isotope abundance is 4.5 times smaller than for natural SiC crystals. Here, the authors should include label axis in Fig. S1, since the factor of 4.5 is not clearly visible without a proper scale. In addition, as the measurements are performed at different temperatures (namely 100K and 300K) for both samples (natural and isotope), the authors should discuss the effect of the temperature, which seems to be crucial for the system.

On the second page, the authors write "Following table I, only the spin quadrupole D0 is excited under these conditions…". Here the authors should clarify and explicitly state which are the exact conditions, as it is not clear from the text.

Also on the second page, the authors state that the simulated data are in contrast with the experimental observation (i.e. "…even when Bz ≠0 …, which contradicts to the experimental observation of Fig 1(c)"). However, the authors do not clarify the reason of such discrepancy, which should be indeed commented.

On page 4, the authors write that they can calculate the effective magnetic field with high accuracy. However, they do not clarify how to extract θ=19° from the data, as this is not obvious. Furthermore, they claim high accuracy, but errors are missing for some values (e.g. θ, ϒ, vprobe), which questions the resulting errors/accuracy of the effective magnetic field. Here, the authors need to include all errors.

In order to improve the quality of the manuscript, I would also suggest some minor revisions.
To make the subject better understandable for a broader audience, as a target of Nature Communications, the authors need to include a longer introduction covering state-of-the-art and alternative approaches that have been used so far. Indeed, it is sufficient to mention them in the abstract.

The authors should comply with the "Nature format" (Introduction – Results – Discussion), as they tend to switch between results and discussion. Furthermore, at some points of the manuscript and supplementary, the English needs to be improved for a better readability.
In the 'Methods', the authors should specify their equipment in more detail (i.e. manufacturer of components and devices, laser linewidths, pump powers, etc.).
In conclusion, after implementing all the proposed changes, the submitted manuscript might fit the

scope and quality of Nature Communications, and might be considered for publication in that journal.

Reviewer #2 (Remarks to the Author):

This paper reports on the manipulation of spin qudit modes based on ensemble of defects in SiC. Although the methods and concepts used in this paper are not particularly new, they are beautifully combined and applied to a rich quantum system (defects in SiC) that is promising for practical applications of quantum technologies, and I think this work has the potential to generate significant interest in the quantum community. The paper is well presented and written (apart from typos), and the data well supported by a theoretical model. For these reasons, I recommend publication in Nature Communications, after the authors addressed the following points:

1) I don't think the title is well chosen, because "sub-MHz resolution" sound very mediocre unless we know the context of the work (SiC defects). I would suggest simply: "Excitation and coherent control of spin qudit modes in silicon carbide" possibly with the addition of "at room temperature".

2) The authors show that their approach enable absolute DC magnetometry (i.e. immune to thermal noise and strain inhomogeneity), but with standard (single drive) ODMR spectroscopy it is easy in general to find two spin transitions that also enable the same thing. Do the authors mean that they can do Ramsey-based absolute magnetometry? It would be useful to clarify this point in the text.

Reviewer #3 (Remarks to the Author):

The authors investigate silicon vacancy defects in SiC as a potential room-temperature qudit. First, they introduce formalism for how the system will behave: for instance, having an independent lifetime for each multipole. Optical pumping polarises the system, which can subsequently be examined by mixing the states with state-selective microwaves. A spectrally-inhomogeneous ensamble is probed. Excitation by microwave manifests as depletion of the fraction of the ensemble that is resonant. ODMR spectra are given (importantly, with dependence on magnetic field). Theory is given and justified. Next, two transitions are controlled. First the \nu_1 transition (-3/2 -> -1/2) is pumped. Then the \nu_5 transition (-1/2 -> +1/2) is pumped. Rabi and Ramsey experiments are done on the m_s = 1/2 doublet, using \nu_1 to prepare the state. Coherence time is measured. Prospects for noise- and inhomogeneity- insensitive DC magnetometry are mentioned.

The technical quality of this work seems high. However, the study relies on ensemble measurements and does not show individual defect centers, which would be interesting. As for the magnetic field sensing application, it does not appear to be competitive against atomic gases or diamond nitrogen vacancy ensembles. I believe this work would fit better in a more specialised journal.

Detailed Points:

White space and formatting in Table 1.
Formalism or citation to justify the optical pumping to either m_s = \pm3/2 or m_s = \pm 1/2 depending upon 'SiC polytype and crystallographic site'?
Discussion of why the two images in figure 2 do not match perfectly? What effect outside your formalism is thought to cause this?
A few grammatical errors in the main text.
In equation S6, D (the zero-field splitting?) is not explicitly defined.
B_0 || z is assumed for equation S14. Do results change significantly if perpendicular components are present? e.g. from the Earth's magnetic field, which were not compensated for?

Comments to reviewers:

Reviewer #1 (Remarks to the Author):

The manuscript "Excitation and coherent control of spin qudit modes with sub-MHz spectral resolution" by V.A. Soltamov et al. deals with the excitation and control of spin qudits based on a silicon carbide platform at room temperature. The background of different polymorphs and the related theory are first discussed, followed by the characterization of the sample by means of optically detected magnetic resonance and electron paramagnetic resonance. Prior to the experimental and numerical part, different broadening mechanisms are examined in order to give a better understanding of the spin qudit spectrum. Finally, simulations and experiments are performed, which demonstrate the excitation of different spin qudit modes, as well as their coherent control by means of Ramsey interferometry.

The submitted manuscript demonstrates new schemes for the coherent control of solid state spin qudits at room temperature, which represents a novelty in the solid-state field. However, before considering acceptance in Nature Communications, I would suggest the following major revisions.

Our response:
We appreciate the recognition of the reviewer of the novelty of the approach in the solid-state field.

Comment 1: On the first page, the authors claim that the 29Si isotope abundance is 4.5 times smaller than for natural SiC crystals. Here, the authors should include label axis in Fig. S1, since the factor of 4.5 is not clearly visible without a proper scale. In addition, as the measurements are performed at different temperatures (namely 100K and 300K) for both samples (natural and isotope), the authors should discuss the effect of the temperature, which seems to be crucial for the system.

Our response:
We thank reviewer for justified criticism. From the spectra shown in Fig. S1 it was not straightforward how to make quantitative conclusions on the isotope abundance. We have therefore revised the corresponding section in the Supplement Material (Methods. Samples), included Equation (S1) and the new spectrum Fig. S1, which allow estimation of the 29Si isotope concentration in our 6H-28SiC sample. The new EPR spectrum in Fig. S1b is shown together with the theoretical spectrum calculated for the 29Si isotope concentration of 1%, which are in a very good agreement. To estimate the 29Si isotope content, we determined the ratio of the intensities of the fine structure EPR transitions (I_FS), corresponding to the transition with I = 0, and the hyperfine EPR transitions (I_HF) due to the hyperfine interaction with the magnetic isotope (I = 1/2 in case of 29Si). We note, the ratio I_HF / I_FS remains unchanged with temperature.

Comment 2: On the second page, the authors write "Following table I, only the spin quadrupole D0 is excited under these conditions…". Here the authors should clarify and explicitly state which are the exact conditions, as it is not clear from the text.

Our response:
We agree, this issue was misleading. We intended to say that optical pumping of SiC results in a preferential population of either the ± 3/2 or ± 1/2 spin state of V_{Si} (depending on SiC polytype and the V_{Si} crystallographic site) and that this spin alignment is theoretically described by the D_0 component of the spin quadrupole. To clarify this issue we have modified the text. Now, it reads
"Optical pumping results in a preferential population of either the $m_S = \pm 3/2$ or $m_S = \pm 1/2$ states (depending on SiC polytype and the V_{Si} crystallographic site, as was shown in Refs. [9, 12, 20, 23]). Such a spin alignment is theoretically described by the contributions to the diagonal components of the spin density matrix δρ+3/2,+3/2 = δρ-3/2,-3/2 = -δρ+1/2,+1/2 = -δρ-1/2,-1/2. In the multipole decomposition of the spin density matrix, this corresponds to the appearance of the spin quadrupole D_0, as shown in the Table I."

Comment 3: Also on the second page, the authors state that the simulated data are in contrast with the experimental observation (i.e. "…even when Bz ≠0 …, which contradicts to the experimental observation of Fig 1(c)"). However, the authors do not clarify the reason of such discrepancy, which should be indeed commented.

Our response:
We consider two most probable sources of inhomogeneous broadening: (i) magnetic fluctuations and (ii) variations of the zero-field splitting around its mean value. The discussion above corresponds to the mechanism caused by magnetic fluctuations. The fact that the simulated data contradict the experimental observation indicates that this mechanism does not play an important role.
We have rewritten this part of the paper in a more accurate way and underlined that theoretically, if the broadening is caused by the magnetic field fluctuations, the frequency of qudit modes doesn't depend on the magnetic field B_z. However, our experiment proves the opposite. Thus, we conclude that magnetic fluctuations are not the main source of inhomogeneous broadening.

Comment 4: On page 4, the authors write that they can calculate the effective magnetic field with high accuracy. However, they do not clarify how to extract ϑ=19° from the data, as this is not obvious. Furthermore, they claim high accuracy, but errors are missing for some values (e.g. ϑ, ϒ, νprobe), which questions the resulting errors/accuracy of the effective magnetic field. Here, the authors need to include all errors.

Our response:
We thank the Reviewer for this comment, because it helped us to recalculate the accuracy of our measurement protocol more precise.

θ is the angle between the magnetic field direction and the c-axis of SiC, and it was obtained from the ratio (ν2 – ν1) / (ν4 – ν3) using the data in Fig. 3(a,b) following the previously described procedure in Ref. 29. It was also shown there, that θ can be determined with a resolution better than 1°. The ν_{probe} frequency is determined with the sub-Hz accuracy as followed from the specification of the RF signal generator. The gyromagnetic ratio γ is the constant value. Taking into account the 0.5 degree deviation of the angle θ from its mean 19 degree value, we have established the accuracy of our measurement protocol to be +/- 2 μT and +/-3 μT. We have added the mentioned above explanations after equation (3) on page 5.

Comment 5: In order to improve the quality of the manuscript, I would also suggest some minor revisions. To make the subject better understandable for a broader audience, as a target of Nature Communications, the authors need to include a longer introduction covering state-of-the-art and alternative approaches that have been used so far. Indeed, it is sufficient to mention them in the abstract.

Our response:
Following this recommendation we have considerably extended the introduction. It covers now state-of-the-art and alternative approaches, including flying and stationary qudits realized in photonic chips, superconducting circuits. We also highlighted the advantages of our approach. The main properties of optically active centers in SiC and its intrinsic properties are also discussed in order to give an overview of recent achievements in this field of quantum technology.

Comment 6: The authors should comply with the "Nature format" (Introduction – Results – Discussion), as they tend to switch between results and discussion. Furthermore, at some points of the manuscript and supplementary, the English needs to be improved for a better readability.
In the 'Methods', the authors should specify their equipment in more detail (i.e. manufacturer of components and devices, laser linewidths, pump powers, etc.).

Our response:
We have significantly rewritten the paper and divided it into appropriate sections: Introduction, Results, Discussion and Methods. The "Methods" section includes now information about the samples used in our experiments (Sample preparation), description of the setup used in our experiments (Two-frequency optically detected magnetic resonance) as well as the theory of multipole spin dynamics (Multipole decomposition), which previously had been in the Supplementary information file. English has been corrected as well.

Reviewer: In conclusion, after implementing all the proposed changes, the submitted manuscript might fit the scope and quality of Nature Communications, and might be considered for publication in that journal.

Reviewer #2 (Remarks to the Author):

This paper reports on the manipulation of spin qudit modes based on ensemble of defects in SiC. Although the methods and concepts used in this paper are not particularly new, they are beautifully combined and applied to a rich quantum system (defects in SiC) that is promising for practical applications of quantum technologies, and I think this work has the potential to generate significant interest in the quantum community. The paper is well presented and written (apart from typos), and the data well supported by a theoretical model. For these reasons, I recommend publication in Nature Communications, after the authors addressed the following points:

Our response:
We appreciate the recognition of the quality of our work and the fact that it is generally suitable for publication in Nature Communications after revision.

Comment 1: I don't think the title is well chosen, because "sub-MHz resolution" sound very mediocre unless we know the context of the work (SiC defects). I would suggest simply: "Excitation and coherent control of spin qudit modes in silicon carbide" possibly with the addition of "at room temperature.

Our response:

We thank the Reviewer for the suggestion to modified the title. Now it reads: "Excitation and coherent control of spin qudit modes in silicon carbide at room temperature"

Comment 2: The authors show that their approach enable absolute DC magnetometry (i.e. immune to thermal noise and strain inhomogeneity), but with standard (single drive) ODMR spectroscopy it is easy in general to find two spin transitions that also enable the same thing. Do the authors mean that they can do Ramsey-based absolute magnetometry? It would be useful to clarify this point in the text.

Our response:
If the ZSF depends on temperature and strain variations, the magnetometry using standard ODMR spectroscopy is not accurate. The equation (3) does not contain ZFS as parameter, therefore, the answer is yes, the Ramsey-based absolute magnetometry is possible and we demonstrate it. In reply to this comment we have added on page 4: "To demonstrate advantages of the qudit modes for quantum information processing and sensing, we demonstrate Ramsey-based absolute magnetometry, performing two-frequency experiments using the protocol presented in Fig. 4(a) (and Supplementary Fig.S2)."

Reviewer #3 (Remarks to the Author):

The authors investigate silicon vacancy defects in SiC as a potential room-temperature qudit. First, they introduce formalism for how the system will behave: for instance, having an independent lifetime for each multipole. Optical pumping polarises the system, which can subsequently be examined by mixing the states with state-selective microwaves. A spectrally-inhomogeneous ensemble is probed. Excitation by microwave manifests as depletion of the fraction of the ensemble that is resonant. ODMR spectra are given (importantly, with dependence on magnetic field). Theory is given and justified. Next, two transitions are controlled. First the \nu_1 transition (-3/2 -> -1/2) is pumped. Then the \nu_5 transition (-1/2 -> +1/2) is pumped. Rabi and Ramsey experiments are done on the m_s = 1/2 doublet, using \nu_1 to prepare the state. Coherence time is measured. Prospects for noise- and inhomogeneity- insensitive DC magnetometry are mentioned.

The technical quality of this work seems high. However, the study relies on ensemble measurements and does not show individual defect centers, which would be interesting. As for the magnetic field sensing application, it does not appear to be competitive against atomic gases or diamond nitrogen vacancy ensembles. I believe this work would fit better in a more specialised journal.

Our response:
We appreciate the Reviewer evaluation of the high technical quality of our work. We also agree, at this stage the sensitivity (i.e., ODMR contract, coherence time) of the so far known spin centers in SiC is lower than that of NV defects in diamonds and atoms. However, the great variety of spin defects and SiC polytypes, along with their technological maturity does no exclude the competitiveness. Additionally, the magnetic field measurement protocol proposed is novel and can be realized only using spin systems with S greater than 1. It should be mentioned that atomic magnetometers provide record sensitivity up to 0.54 fT*$Hz^{-1/2}$ with a measurement volume 0.3 cm^3 [Kominis et al., Nature **422**, 596–599 (2003)], thus suffering by the low spatial resolution. Magnetometers, based on single NV center in diamond allows magnetic field measurements with sensitivity lower than that of atomic magnetometers, but with spatial resolution in nm range [Zopes et al., Nature Communications **9**, 4678 (2018)]. However, there is a variety of applications where such high sensitivity and spatial resolution are not necessary. In these cases, magnetometers based on our approach can be used. Additionally, due to low developed level of the diamond manufacturing and peculiarities of the NV center ground spin state, magnetometry based on the NV centers is sample dependent. Our approach demonstrates the sample-independent magnetometer on a cheap and robust platform, such as SiC. Unlike diamond, SiC is well developed being a mature semiconductor material. We would like to emphasize that the magnetometry is not the only one massage of the paper. The central result is that we demonstrate the usability of V_{Si} centers in SiC as a room temperature qudit. The latter is impossible to realize on NV centers in diamond.

Comment 1: Detailed Points: White space and formatting in Table 1.

Our response:
We have decided to postpone this until a later date, as all tables will be reformatted by the technical editor in the production phase. At this stage we only checked the accuracy of the content.

Comment 2: Formalism or citation to justify the optical pumping to either m_s = \pm3/2 or m_s = \pm 1/2 depending upon 'SiC polytype and crystallographic site'?

Our response:
We have added the corresponding references. Namely, refs. 9, 12, 20, 23.

Comment 3: Discussion of why the two images in figure 2 do not match perfectly? What effect outside your formalism is thought to cause this?

Our response:
Some discrepancy between the measured and calculated images may stem from more general form of spin relaxation, not described by a single parameter, or more general form of inhomogeneous broadening. Introducing more parameters in theory will definitely enable one to obtain a better fit. However, we would like to avoid multiplying the number of fit parameters since their determination is not reliable. We have added the corresponding comment on page 4, at end of the second paragraph.

Comment 4: A few grammatical errors in the main text.

Our response:
Errors and typos found in the text have been corrected.

Comment 5: In equation S6, D (the zero-field splitting?) is not explicitly defined.

Our response:
We hope we clarified this issue in a revised version of our manuscript. The theoretical part has been removed from the Supplemental material file. Now it is placed in the main text of the paper (please, see "Methods" section and "Multipole decomposition" therein). So the Eq. S6 became Eq. 9. We have added the following below the Eq. 9: "2D is the zero-field splitting".

Comment 7: "B_0 || z is assumed for equation S14. Do results change significantly if perpendicular components are present? e.g. from the Earth's magnetic field, which were not compensated for?"

Our response:
In Eq. (S14) [Eq. 15 in the new version], we present the matrix of spin relaxation rates for B||z, which has a rather compact form and can be used to qualitatively explain why some of the modes are not visible for certain relation between relaxation times of Tp, Td, and Tf. In the further calculations, leading to Fig. 2b, we use the general expression of Eq. (S13) [new version Eq. 14] for the spin-relaxation-rate matrix accounting for the perpendicular magnetic field components.

REVIEWERS' COMMENTS:

Reviewer #1 (Remarks to the Author):

The authors of the manuscript entitled “Excitation and coherent control of spin qudit modes in silicon carbide at room temperature” have properly addressed all our comments and revised the paper accordingly. Therefore, the manuscript can be now recommended for publication in Nature Communications”

Reviewer #2 (Remarks to the Author):

The authors satisfactorily addressed the points raised by the referees and I recommend publication in Nat. Comm.

Reviewer #3 (Remarks to the Author):

The authors have addressed my technical questions. From a technical perspective, I believe the work is valid and of high quality. However, given frequency of reports about quantum emitters, and the fact that the reported values are not better than what has already been presented in other systems, I still believe that this work would be better suited for a more technical journal, such as PRB.

AUTHOR‘S RESPONSE TO REVIEWERS’ COMMENTS:

REVIEWERS' COMMENTS:

Reviewer #1 (Remarks to the Author):

The authors of the manuscript entitled “Excitation and coherent control of spin qudit modes in silicon carbide at room temperature” have properly addressed all our comments and revised the paper accordingly. Therefore, the manuscript can be now recommended for publication in Nature Communications”

We thank the reviewer for his/her final recommendation to publish the paper in Nature Communications.

Reviewer #2 (Remarks to the Author):

The authors satisfactorily addressed the points raised by the referees and I recommend publication in Nat. Comm.

We thank the reviewer for his/her final recommendation to publish the paper in Nature Communications.

Reviewer #3 (Remarks to the Author):

The authors have addressed my technical questions. From a technical perspective, I believe the work is valid and of high quality. However, given frequency of reports about quantum emitters, and the fact that the reported values are not better than what has already been presented in other systems, I still believe that this work would be better suited for a more technical journal, such as PRB.

We appreciate the recognition of the quality and validity of our work by the reviewer and his/her recommendation to publish it in the PRB, which in our opinion is a high-ranking physics journal. With regard to his/her skepticism, we dare to claim that our work is not just one more technical report on quantum emitters, but discusses the fundamentals of several possible applications of less common spin qudit modes for quantum information processing, sensor technology and magnetometry - all by performing a two-frequency protocol at room temperature. To the best of our knowledge, the spin qudits are difficult to implement, e.g. with NV centers in diamonds, but in SiC they seem to be possible.
